# Genetics of Lipodystrophy: Can It Help in Understanding the Pathophysiology of Metabolic Syndrome?

**DOI:** 10.3390/biom8030047

**Published:** 2018-07-06

**Authors:** Sandeep Kumar Mathur, Pradeep Tiwari, Sonal Gupta, Nidhi Gupta, Surendra Nimesh, Krishna Mohan Medicherla, Prashanth Suravajhala

**Affiliations:** 1 Sawai Man Singh Medical College and Hospital, JLN Marg, Jaipur 302004, India; 2 Department of Biotechnology and Bioinformatics, Birla Institute of Scientific Research, Statue Circle, Jaipur 302001, India; tiwari.pradeep30@gmail.com (P.T.); gupta.sonal1990@gmail.com (S.G.); kmohan@bisr.res.in (K.M.M.); 3 Department of Biotechnology, IIS University, Mansarovar, Jaipur 302020, India; nidhinimesh@gmail.com; 4 Department of Biotechnology, Central University of Rajasthan, Bandarsindri, N.H. 8, Kishangarh 305801, India; surendranimesh@gmail.com

**Keywords:** lipodystrophy, metabolic syndrome, type-2 diabetes mellitus, obesity, regulation, functional genomics, systems biology, phenotypic traits, insulin resistance

## Abstract

Understanding phenotypes and their genetic determinants for metabolic syndrome (MetS) has been quite challenging. With the advent of systems genomic approaches, there is a need to decipher methods for identification and evaluating the functional role of phenotypic traits associated with complex diseases, such as MetS. The monogenic syndromes of lipodystrophy are well understood, but the molecular pathophysiology of insulin resistance (IR) underpinning the obesity, diabetes mellitus, and dyslipidemia is not well deciphered. In this commentary, we argue the role of pathophysiology of MetS, and its effects into possible understanding of genetic determinants associated with lipodystrophy-mediated diabetes mellitus.

## 1. Introduction

Adipose tissue stores triglycerides to sustain the metabolic activities in cells. When it does not, this leads to monogenic disorders or complex metabolic disorders of insulin resistance (IR) like diabetes mellitus, dyslipidemia, hypertension, etc. [1]. Although the pathogenic mechanism in monogenic syndromes of lipodystrophy is well understood [2], the molecular pathophysiology of IR underpinning the obesity, diabetes mellitus and dyslipidemia metabolic syndrome (MetS) is not well deciphered [3]. Currently, we are in the era of global pandemic of MetS [4], and recent advances from cellular and molecular investigations suggest the role of poor adipogenesis and consequent ectopic fat deposition as one of the major pathophysiological mechanisms of IR. Further, type-2 diabetes, in relation to monogenic diseases such as maturity onset diabetes of the young (MODY), lipodystrophy, and their phenotypic traits, has been recently documented [5]. Therefore, lipodystrophy could be considered as a relevant model for studying molecular pathophysiology of MetS. In addition, genome-wide association studies (GWAS) have yielded a number of mutations, augmenting the fact that there are lipodystrophy variants contributing to obesity and diabetes mellitus [6,7]. 

## 2. Discussions

Understanding the molecular mechanism and pathophysiology of MetS is expected to generate enormous scope to treat non-monogenic diseases, like obesity, on the lines of monogenic disorders like lipodystrophies. For example, lamin A/C (*LMNA*) is associated with Dunnigan-type familial partial lipodystrophy (*FPLD*; OMIM 151660) which is a rare monogenic form of IR [8]. In addition, AKT serine/threonine kinase 2 (*AKT2*), 1-acylglycerol-3-phosphate O-acyltransferase 2 (*AGPAT2*), and peroxisome proliferator activated receptor gamma (*PPARG*) equally contribute to the metabolic dysfunction of these diseases [9]. While *AGPAT2* is known to be associated with Berardinelli–Seip congenital lipodystrophy (BSCL) syndrome, it is characterized by the absence of fat predisposition to develop diabetes mellitus, and further correlates with mutational spectrum forming phenotypic heterogeneity [10]. Interestingly, from our recent systems phenome-interactome studies, these three genes are among a host of differentially expressed genes (DEGs) known to be enriched and associated with the phenotypic traits [11]. 

**Figure 1:**
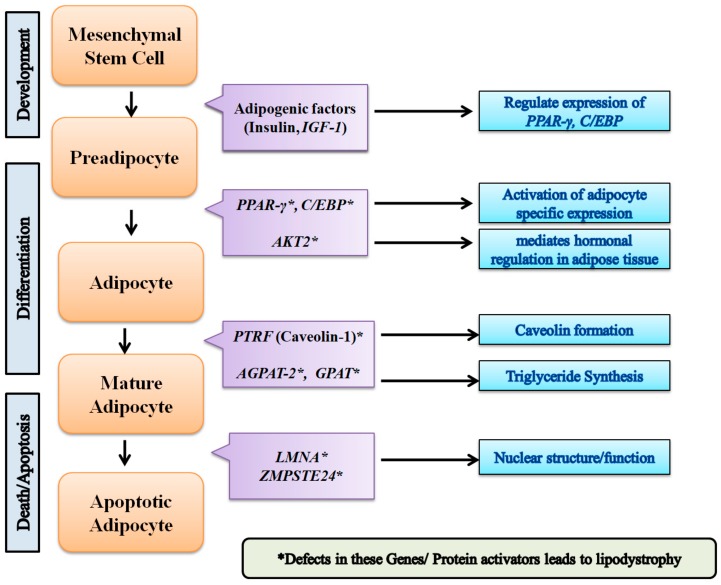
Association of lipodystrophy-associated genes and their activators at various phases of adipogenesis. *IGF-1*: insulin like growth factor 1; *PPAR-γ*: peroxisome proliferator activated receptor gamma; C/EBP: CCAAT/enhancer binding protein; *AKT2*: AKT serine/ threonine kinase 2; *PTRF*: polymerase I and transcript release factor; *AGPAT-2*: 1-acylglycerol-3-phosphate o-acyltransferase 2; *GPAT*: glycerol-3-phosphate acyltransferase; *LMNA*: Lamin A/C; *ZMPSTE24*: zinc metallopeptidase STE24.

**Figure 2:**
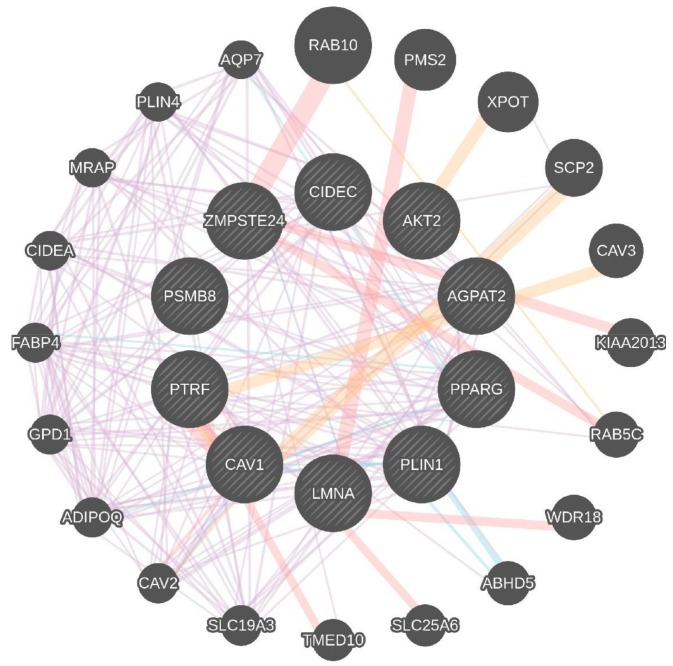
Systems network of Lipodystrophy specific genes in bigger circles. The lipodystrophy-related genes were subjected to GeneMANIA [17], a network prediction database, and we visualized the genes responsible for lipodystrophy to form a network with other genes. The pink edges show that they are physically interacting which in this case all central nodes (genes) are shown to have experimental (physical) interactions, while the other edges show that they are localized to the same organelle (blue edges connected by nodes, *viz. CAV1* and *CAV2*; *ZMPSTE24* and *LMNA*; *SCP2* and *AGPAT2*). The list of lipodystrophy related genes is tabulated in Table 1.

The genes that are known to play a role in adipocyte differentiation and regulation [12] are also known to be involved in number of metabolic pathways of glucose and lipid homeostasis under healthy physiological conditions, disorders of which could potentially be associated with MetS (Figure 1). We argue that intervention of additional phenotypic traits can be used to study the novel pathways and genes implicated in the occurrence of monogenic diseases which play an important role in development and death of adipocytes. For such associations, we can take measure of these phenotypic traits and predict the possibility of risk factors in those subjects, as they could be predictors for long-term cardiovascular risk [13]. MetS, as a complex trait, along with several other intermediary phenotypic traits as clusters, delves into possible understanding of reproducible genetic markers from these studies. Although characteristic clinical phenotypes, such as body mass index (BMI) statistics, adipocyte size, lipid parameters, homeostatic model assessment-insulin resistance (HOMA-IR), beta cell function HOMA-β, etc., are in use, genomicists are still in process of acquiring the deep phenotypes to study phenome-interaction studies. In addition, monogenic disorders of MODY diabetes, mitochondrial diabetes would be interesting to look for the pathways resembling classes of MetS, which will allow us to understand recapitulation of key clinical phenotypic traits. Given the heterogeneity of lipodystrophies and the mutated genes leading to metabolic and endocrine dysfunction, one can expect complete or partial IR in subjects. When we subjected all lipodystrophy-related genes to a network, a great deal of pathology is associated with *LMNA* gene, providing clues about pathways for genetic determinants of diabetes mellitus and subtypes based on unique lipodystrophy related pathways (Figure 2). From our analyses, we observed a number of DEGs specific to subcutaneous and visceral adipose tissue compartments [11]). While tumor progression locus 2 (*TPL-2*), *LMNA*, chloride intracellular channel 1 (*CLIC-1*), hypoxia inducible factor 1 (*HIF-1*), six transmembrane epithelial antigen of the prostate 4 metalloreductase (*STEAP-4*) and cell death inducing DFFA like effector c (*CIDEC*) have been known to be observed in subcutaneous adipose tissue-related pathways, mitochondrial calcium uniporter (*MCU*) and leptin (*LEP*) are known to be associated with pathways inherent to visceral adipose tissue [14]. On the other hand, genes specific to lipodystrophy like insulin-like growth factor II (*IGF2*) was shown to play a role in differentially methylated region (DMR) [15]. The quantitative traits indicate that the methylation mark is consistently seen up to the middle ages, with their effect on environment in human development possibly detected many years later. One such epigenetic deregulation by *LMNA* mutations was recently shown [16]. Given the lack of epigenomic studies correlating these genes specific to lipodystrophy, there is a need to understand the influence of genes in the environment. For this, systems genomics could possibly provide additional theories to address this.

## 3. Conclusions

There is a need to discern candidate genes and mutations in not just monogenic diseases, but polygenic traits, like MetS. We are in need of identifying deep phenotypic traits responsible particularly for the MetS and develop phenome-interactome studies inferring the causality; (b) finding pathway correlation to infer genetic determinants of the diseases that could serve as characteristic biomarkers; and (c) considering the phenotypic traits of monogenic disorders and MetS, taken together, which can open avenues for understanding novel therapeutic strategies.

## Figures and Tables

**Table 1:** The list of lipodystrophy related genes and their possible role in MetS, as queried using GeneMANIA (Figure 2).

Key Genes	Gene Function
*AGPAT2*	*AGPAT2* catalyzes formation of phosphatidic acid.
*AKT2*	*AKT2*, also known as protein kinase B. Involved in adipocyte differentiation. Downstream insulin receptor signaling.
*CAV1*	Caveolin 1 binds and transports fatty acids to lipid droplets.
*LMNA*	Lamins A and C are nuclear lamina proteins linked with the cytoskeleton.
*PLIN1*	Perilipin 1 is an essential component for lipid storage.
*PPAR-γ*	*PPARG* is crucial for adipogenesis and for maintenance of the differentiation phase.
*PTRF*	Creates caveolae and regulates expression of caveolins 1 and 3.
*ZMPSTE24*	A zinc metalloproteinase involved in the correct processing and maturation of lamin A (*LMNA*).
*CIDEC*	A cell death-inducing DNA fragmentation factor-like effector family with important roles in apoptosis, regulated by insulin and its expression is positively correlated with insulin sensitivity.
*PSMB8*	A proteasome subunit beta type-8 as known as 20S proteasome shapes the antigenic repertoire presented on major histocompatibility complex (MHC) class I molecules.
